# A thesaurus of genetic variation for interrogation of repetitive genomic regions

**DOI:** 10.1093/nar/gkv178

**Published:** 2015-03-27

**Authors:** Claudia Kerzendorfer, Tomasz Konopka, Sebastian M.B. Nijman

**Affiliations:** Research Center for Molecular Medicine of the Austrian Academy of Sciences (CeMM), Vienna, Austria

## Abstract

Detecting genetic variation is one of the main applications of high-throughput sequencing, but is still challenging wherever aligning short reads poses ambiguities. Current state-of-the-art variant calling approaches avoid such regions, arguing that it is necessary to sacrifice detection sensitivity to limit false discovery. We developed a method that links candidate variant positions within repetitive genomic regions into clusters. The technique relies on a resource, a thesaurus of genetic variation, that enumerates genomic regions with similar sequence. The resource is computationally intensive to generate, but once compiled can be applied efficiently to annotate and prioritize variants in repetitive regions. We show that thesaurus annotation can reduce the rate of false variant calls due to mappability by up to three orders of magnitude. We apply the technique to whole genome datasets and establish that called variants in low mappability regions annotated using the thesaurus can be experimentally validated. We then extend the analysis to a large panel of exomes to show that the annotation technique opens possibilities to study variation in hereto hidden and under-studied parts of the genome.

## INTRODUCTION

Detection of genetic variation is one of the main applications of high-throughput sequencing and several software solutions exist tailored to this task (1,2). These methods have already enabled breakthroughs in understanding of cancers (3–5). They have also helped diagnosis in clinical case studies (6). Hospitals are thus considering more widespread use of sequencing technologies to inform treatment of patients (7,8). However, efforts to limit the false discovery rate during variant calling have led bioinformatic methods to avoid analyzing regions of the human genome where alignment of short reads poses ambiguities. Thus, despite abundance of raw data, much genetic variation in such regions still remains uncharacterized.

Low-mappability regions are segments of a genome that are identical, or almost so, to other segments. The term has been used to describe between 10 (9) and 50% (10) of the human genome. Even the conservative definitions include tandem repeats, transposable elements, portions of genes (some of which linked to human disease, e.g. MLL3 to leukemia and IKBKG to immunodeficiencies), and substantial portions of entire gene families (e.g. >90% of sequence in HLA and PAR1 gene families). Avoidance of low mappability regions during variant calling or variant candidate selection thus hides information about genetic variation relevant for human disease. It obscures the view of heterogeneity in cancer. It may also, in part, explain why studies of patients with suspected Mendelian disease achieve imperfect diagnosis rates (25% in (7)).

The difficulty with analyzing variants in low mappability regions using short (e.g. 100-bp single-end or paired-end reads) can be illustrated via the following example. Suppose that a sequence pattern is present at two different locations in the reference genome and that a sample contains a single-nucleotide variant (SNV) in one of these regions. Upon library preparation and sequencing, the variant is encoded in short reads, which do not carry information about their broader context. Thus, the reads are not uniquely mappable to the reference genome. Mapping software can either distribute them randomly across both mapping sites or report more than one alignment. But regardless of the mapping strategy, reads with the mismatch end up positioned across more than one genomic site and labeled with a low mapping quality. A related difficulty appears again during variant calling. On the one hand, ignoring mapping quality leads to calls for both sites and over-estimates the degree of genetic variation in the sample. On the other hand, ignoring the sites altogether leads to false negatives (FNs). Thus, any ‘local’ variant analysis method—a method that considers only one genomic site at a time or that reports variants at single sites—is prone to imperfection when working with low mappability regions.

As illustrated by the example, mappability affects variant detection starting at the stage of read generation, through alignment, and up to candidate selection. Sequencing with long reads would reduce the fraction of the genome affected by low mappability. Length can be achieved in the physical sense, e.g. from Sanger or other technologies, or in the logical sense, e.g. using molecule bar-coding after proximity ligation (11) or dilution fragmentation (12,13). However, these techniques are more expensive and/or require more laborious library preparation than shotgun sequencing, so their suitability for large-scale studies remain limited. The logical long read protocols have not been used on heterogeneous samples, they have not been coupled with enrichment strategies for exomes or other gene sets. They also require analysis methods based on genome assembly, which are more intensive than alignment based methods. Although these obstacles may be overcome in the future, computational methods will nonetheless be important to utilize the large amounts of already existing short-read data.

Tools such as Sniper (14) and others (15) already addressed some of the difficulties associated with repetitive regions and short-read data. They showed that coupling re-alignment of select reads with models of expected coverage can improve calling sensitivity. However, these approaches re-process entire datasets starting from the raw unaligned input. This entails a considerable computational cost, part of which is spent on duplicating work already performed by established tools. Furthermore, these approaches strive to report variants at individual sites, which as explained above, is inherently prone to imperfection in genomic regions of high similarity.

In this work we set out to detect and annotate variants from short-read data without reprocessing or duplicating data, changing storage formats or rewriting entire analysis pipelines. We designed a resource—a thesaurus of genetic variation—for quick look-up of similar genomic regions. Because this resource contains precomputed multi-mapping locations, we need not repeat similar calculations for every read in every sample. Instead, we use the resource to annotate already called variants, for example linking them into clusters. The algorithm is asymptotically and practically computationally efficient. It thus enables, to our knowledge for the first time, extensive study of variation in repetitive regions in large datasets.

In the next section, we describe our strategy for building a thesaurus of genetic variation and annotating variants. In the ‘Results’ section, we benchmark the method on simulated datasets to characterize the properties and benefits of the annotations. We also apply the method on whole genome sequencing data from a human cell line, characterizing the properties of annotated variants and validating several candidate hits. Finally, we perform a meta-analysis of a panel of exomes from breast cancer cell lines and healthy human individuals.

## MATERIALS AND METHODS

### Concept

**Figure 1.**
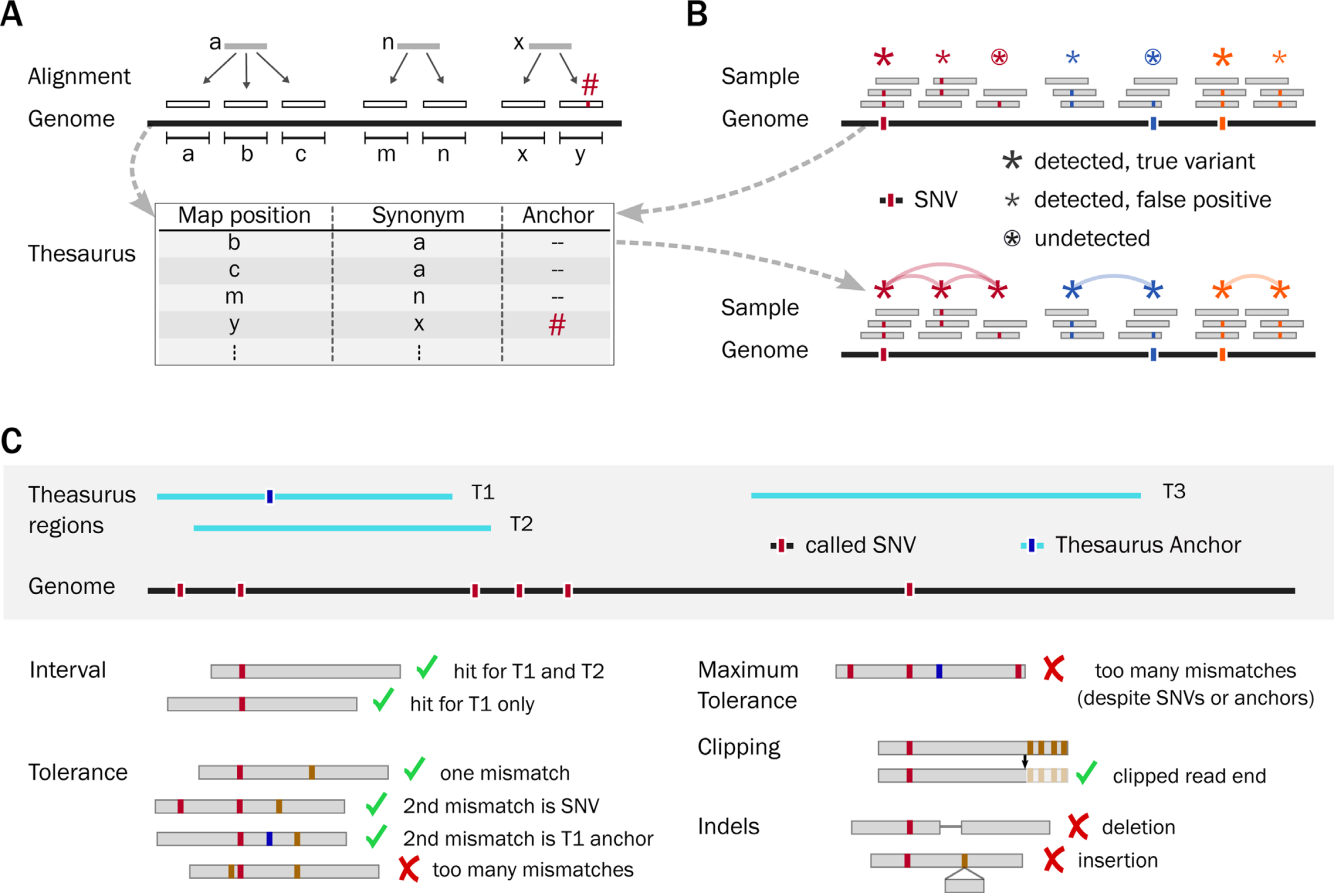
Schematic outline for a thesaurus of genetic variation. (**A**) Scheme for thesaurus creation: on top, reads from regions a, n and x are shown to map onto multiple loci in the reference genome; below, the mappings are collected into a data structure linking pairs of regions, alongside any mismatches. (**B**) Scheme for thesaurus usage: on top, mapped reads show evidence of variants on true as well as false locations; below, similar variants are grouped into clusters using thesaurus annotations. (**C**) Criteria used to associate variants with alternative sites. On top, thesaurus regions and called variants are displayed along a genome. On bottom, reads are compared with thesaurus entries to determine if their mismatch patterns and alignment positions are consistent with a thesaurus link. A link is declared if several reads aligned onto a locus are consistent with the thesaurus annotation.

The intention of a thesaurus of genetic variation is to store links between similar genomic regions (Figure 1A) and to provide a means to use these links to annotate variant calls (Figure 1B). In this section we explain how such links can improve variant analysis through a series of examples.

An illustrative genome may contain stretches of sequence more than once with some SNVs. As a result short reads from shotgun sequencing can be aligned onto the genome, but cannot be assigned uniquely to the true site of origin (alignment, top of Figure 1B). In other words, reads with evidence for genetic variants are distributed over several loci. This causes traditional variant calling approaches to produce either false positives (FPs) calls, FNs calls or both (variant calls, top of Figure 1B). The purpose of a thesaurus is to identify and document such cases and enumerate similarities between called variants and alternative genomic sites (illustrated by the arches, bottom of Figure 1B). These links self-organize into clusters that better reflect the nature of the true variants than the raw variant calls themselves.

For example, evidence for one true variant may distribute over two distinct loci (Figure 1B, orange markers). When both of them are called in a traditional variant call set, this results in one true positive (TP) and one FP. A cluster of links between these two sites, in contrast, suggests there may only be one true change in the genome with respect to the reference. The annotation thus reduces the number of FPs.

In another example, evidence for one variant may again distribute over two sites, but be reported only at the wrong one (Figure 1B, blue markers). This results in one FP and one FN. A thesaurus link between the sites again suggests there is a single variant and here further suggests that it may be located at either of the two locations. Since the annotation links the reported site to the true variant locus, the annotation reduces the numbers of FN as well as FP calls.

In a final example, evidence for a variant may distribute over three loci and be reported at two sites, including the correct location (Figure 1B, red markers). Links from the correct site to an alternative site that is in the call set was covered by a previous example. A link to an alternative site that is not even in the call set may seem like a false alarm—such a link does not alter the number of FP nor FN variants. Nonetheless, the link may still provide meaningful information in that it may point to reads with evidence for the variant.

To summarize, in these examples the illustrative genome as three true variants and the initial call set has five candidates variants (Figure 1B). In a variant analysis based on local information there are two TP, three FP and one FN. Assuming the genome has length 1000, this gives a false positive rate (FPR),

\begin{equation*}
[{\rm FPR}] = [{\rm FP}]/([{\rm FP}] + [{\rm TN}]),
\end{equation*}

around 3/1000 and a true positive rate (TPR)

\begin{equation*}
[{\rm TPR}] = [{\rm TP}]/([{\rm TP}] + [{\rm FN}])
\end{equation*}

equal to 2/3.

In an analysis using thesaurus annotations, the accounting must necessarily be a little different. First, we modify the definitions of FN and FP. We define a FN as a site with a true variant that is not called itself and to which no called variant links to. We define a FP as a site for which the site itself nor any of the sites it may link to correspond to a true variant. Second, we introduce the concept of a thesaurus true positive (TTP), i.e. a called site that is not itself the locus of a variant, but that is linked via a thesaurus link to a true variant. Third, we define the concept of a thesaurus true positive rate (TTPR) as:

\begin{equation*}
[{\rm TTPR}] = ([{\rm TP}] + [{\rm TTP}])/([{\rm TP}] + [{\rm TTP}] + [{\rm FN}])
\end{equation*}

This is an imperfect measure of performance, but it has the desirable property that it reduces to the conventional TPR when TTP = 0. For the examples above, with the thesaurus annotations and above redefinitions, the FPR drops to zero because all called variants are linked with a true site. The TTPR rises to a perfect unity because there are no FNs. The two sites that would be called FPs in the previous analysis now become thesaurus true positives.

### Implementation

We now turn to the implementation of the thesaurus concept. To create the thesaurus resource, we generated a set of error-free 100-bp reads covering the entire human reference genome (hg19) at regular 10-bp intervals. We then considered each read and computed all possible alignments onto the genome (Figure 1A). Although several multi-mappers are available, for example BLAT (16), they are not tailored to systematic whole-genome analysis. We therefore generated mappings with a custom tool. We looked for alignments that did not contain any insertions or deletions, did not contain more than four mismatches and did not contain any mismatches in the first and in the last five bases. We set the last constraint because we used those terminal bases as the only seeds for alignment. Although mismatches in those terminal bases create some FN mappings, this does not frustrate the approach as a whole because these mappings are recovered in later stages from alignments of nearby reads.

We scanned the alignment files and generated a table enumerating pairs of similar genomic regions (Figure 1A). In this step, we limited attention to three mismatches and recorded their positions. We sorted the output to enable look-up of information from the table via streaming. We merged entries together to define longer regions of similarity and thereby reduce the overall size of the table. We also extended regions down- and upstream to account for non-complete sampling in the previous stage and to recover missed alignments due to imperfect seeding.

In its final form, we call the resulting table a thesaurus of genetic variation. The version used in this work for the human genome (hg19) consists of 2.99 × 10^9^ entries assembled from 1.44 × 10^10^ non-trivial alternate alignments of 2.90 × 10^8^ reads. It is an unbiased and annotation-independent representation of non-unique sequence in the human genome.

Next, we implemented a filtering/annotation strategy for genetic variants. In broad terms, thesaurus filtering scans a set of called variants and links some of them to alternative sites. In practice the strategy is based on a combination of conditions and rules. The inputs to the analysis consist of a thesaurus table, a set of called variants and read positions from an alignment (Figure 1C and software documentation). When determining alternate variation sites, we checked each read for overlap with a thesaurus interval and we tolerated some mismatches that may be due to sequencing errors or nearby genomic variants (variants near the annotated site and near the putative alternative sites). We linked a variant to an alternative site if the number of reads and the fraction of reads holding the variant satisfied corresponding thresholds. As part of filtering, we introduced a vocabulary to mark certain subclasses of variants. As the thesaurus table does not contain information about divergences other than substitutions, we marked variants supported by reads containing a large number of errors or any number of deletions, insertions or splice junctions. As some variants were in practice linked to an excessive number of alternative sites, we also marked these variants rather than reporting all the alternate possibilities. Marked variants were removed in downstream analysis in all the examples in this work.

The computational complexity of assigning links to variants is *O(d t v log v)*, where *d* is the average depth of the alignment, *t* is the average number of thesaurus entries associated with a locus and *v* is the number of called variants. The logarithmic factor appears because the algorithm looks for variants near putative alternate sites, which involves search in the set of called variants. Since *t* is fixed and typically not very large, the algorithm is asymptotically fast. In practice, thesaurus filtering may require from half an hour (exome dataset) to a few hours (whole-genome dataset) of compute time on a modern CPU (Supplementary Text section 1).

## RESULTS

### Benchmarking

To quantify the impact of mappability on local variant calling and to measure improvements due to thesaurus annotation, we performed benchmarking and exploratory calculations on synthetic data.

We created a custom genome based on hg19 containing SNVs placed randomly at a rate of one event per kilobase. We then generated synthetic data in single-end and paired-end formats by sampling from the custom genome at regular intervals. We thus obtained datasets with 10× coverage in which all variants have unit allele frequency and are covered by 10 reads. This is convenient because any false or missed sites appearing in variant analysis can be attributed to mappability alone, i.e. not to other complex sequencing artefacts and biases that complicate interpretation of real data (17).

**Figure 2.**
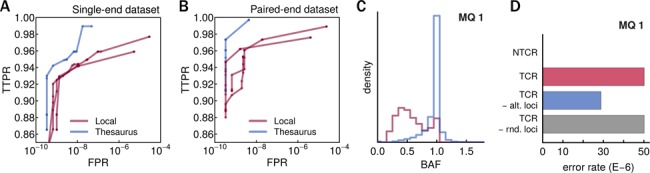
Proof of concept for thesaurus variant annotation. (**A**) ROC-style representation of calling performance on a synthetic dataset with single-end reads. Lines represent performance of three ‘local’ variant callers (GATK, Varscan and Bamformatics) and performance obtained after thesaurus annotation. TTPR stands for Thesaurus True Positive Rate (Methods), FPR stands for False Positive Rate (we assumed the number of true negatives in the human genome was 3 × 10^9^). (**B**) Similar to A, but showing results using paired-end data. (**C**) Distribution of B-allele frequencies attributed to thesaurus-annotated variants; color coding as in previous panels. (**D**) Error rates on genomic regions. NTCR stands for non-thesaurus-covered regions, i.e. regions not described within the thesaurus resource. TCR stands for thesaurus-covered regions. TCR-alt.loci stands for TCR regions minus loci identified as alternate sites to called variants. NTCR-rnd.loci stands for TCR regions minus some randomly selected sites (error calculation repeated five times, error bars are too small to see).

We mapped the synthetic reads onto the reference genome with a fast aligner, Bowtie2 (18). We then called variants with a series of mapping quality thresholds using three variant callers, GATK (19), Varscan (20) and Bamformatics (http://sourceforge.net/projects/bamformatics/). As expected, raw results showed a trade-off between specificity and sensitivity in both single-end and paired-end datasets (Figure 2A, B, Supplementary Text sections 2 and 3). Importantly, the performance of the three callers was similar, indicating that they handle ambiguities in mappability in a fundamentally similar manner. For the next analyses, we therefore continued with just one variant caller. We picked Bamformatics as it was the fastest and also produced the largest number of candidates at the lowest mapping quality thresholds.

We post-processed sets of local variant calls using the thesaurus and obtained links between variants in low mappability regions with possible alternative sites. Sensitivity and specificity were both improved by this procedure (Figure 2A and B). At conservative mappability thresholds, annotation of the called variants only eliminated a small number of FPs. When annotating call sets obtained with low mapping quality thresholds, however, the annotation simultaneously halved the FNR and decreased the FPR by orders of magnitude. As a control, we repeated the analysis using randomly generated links. Such randomly generated links did not yield substantial performance change over non-annotated call sets (Supplementary Text sections 2 and 3).

Next, we explored properties of annotated variants and their alternate loci. Since the benefits of thesaurus annotation were most pronounced when working with low mapping quality thresholds, we here summarize results obtained with this setting (see also Supplementary Text sections 2 and 3). We observed that several of the called sites were linked together, i.e. into clusters. The size of these clusters was rarely >3. The annotations also created links to many uncalled sites: the majority of annotated variants had ten links or fewer, but some had several hundred links (Supplementary Text sections 2 and 3).

Next, we used sites found during thesaurus annotation in calculations of B-allele frequencies (BAFs). When we estimated BAFs using only the reads at the called positions in low mappability regions, we found they were often smaller than unity. This is incorrect since the synthetic genome was haploid. When we evaluated BAF by averaging reads over all alternate loci, however, we obtained better estimates for these variants (Figure 2C).

We also noted that accounting for alternate loci decreased the apparent sequencing error rate (Figure 2D). To evaluate error rates, we counted mismatches in reads at sites that were not labelled as variants (Supplementary Text sections 2 and 3). In low-mappability regions of the genome, the apparent error-rate was three orders of magnitude higher than in high-mappability regions. When we excluded sites linked to called variants, the error rate decreased. As a control, we repeated the same calculation excluding a matched number of randomly selected sites, but we did not observe a similar decrease. These results show that the alignments contained reads bearing true variants at several of the annotated sites. The error estimates did not go to zero because some variants still remained undiscovered (Figure 2A and B).

As a final stage of validation, we explored the impact of alignment on thesaurus filtering. We did not attempt to systematically compare alignment tools on all metrics. Rather, we only investigated elements relevant to thesaurus annotation. Replacing Bowtie2 mapping software by GSNAP (21) repositioned some reads in the alignment and thus primed the variant callers to detect different sets of variants. The new aligner also assigned different mapping quality scores and thus changed the interpretation of our thresholds. We reasoned that the latter problem would be most pronounced at intermediate levels of sequence divergence and least pronounced in highly repetitive regions. We thus focused our attention on calls obtained with low mapping quality thresholds (mapping quality 1). We found ∼5% discrepancy (ratio of discordant to concordant sites) with the original variant calls. Accounting for alternate sites found by thesaurus filtering reduced the discrepancy to ∼2%. This improvement shows that many discrepant calls are due to mapping implementation and that they can be resolved using thesaurus annotation. Upon manual inspection of the remaining discrepant variants, we noted that variants called from one alignment were often visible in the other. Discrepancies were due to a lower number of reads mapped onto the locus or strand-bias induced by misalignment. This suggests that variant detection performance can be pushed beyond the levels reported here (Figure 2) by tuning mapping and variant calling settings prior to thesaurus filtering.

### Application to a haploid genome

We next turned to analysis of a real whole-genome dataset. We chose a paired-end 2 × 100-bp Illumina sequencing dataset with ∼30× coverage from KBM7 (SRX610959, SRX610987). KBM7 is a human leukemic cell line that has previously been described as haploid except for a diploid chromosome 8 and a diploid segment on chromosome 15 (22). The ploidy of most chromosomes makes this dataset a natural extension of the previous calculations based on the synthetic genome.

**Figure 3.**
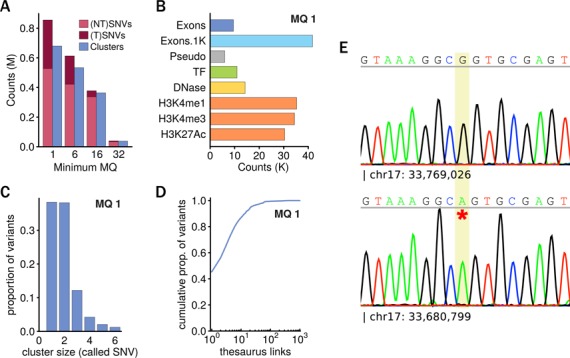
Thesaurus analysis of genome sequencing data from a haploid cell line. (**A**) Variants called with several mapping quality (MQ) thresholds were thesaurus-filtered and grouped into clusters. (T)SNVs and (NT)SNVs refer to sites that were or were not annotated with links to alternate sites, respectively. Variants that were not thesaurus-annotated and were common to all call sets were excluded from the plot (2.5M variants). (**B**) Many thesaurus-annotated variants called with lenient MQ threshold fall in candidate functional regions as defined by ENCODE (19). Exons: exonic regions defined by Gencode (V19); Exons.1K: exonic regions plus 1 kb flanking regions; Pseudo: exonic regions labeled by PseudoGene; TF: transcription factor binding sites in various cell types; DNase: DNase hypersensitivity clusters in various cell types; H3K4me1: histone methylation mark in seven cell types; H3K4me3: histone trimethylation mark in seven cell types; H3K27Ac: histone acetylation marks in seven cell types. (**C**) Cluster sizes formed by called exonic variants linked together by thesaurus annotation. (**D**) Number of alternate sites associated with called variants in exons. (**E**) Two Sanger traces produced by different specific primers. Top trace shows a wild-type sequence within gene SLFN13; bottom trace shows sequence within gene SLFN11 with a homozygous variant at the indicated position.

We started the analysis with a conservative set of calls and then repeated the analysis with lower mapping quality thresholds. Approximately 2.5M sites were common to all call sets and, as expected, reducing the threshold increased the number of identified variants (Figure 3A). After thesaurus annotation, several variants called at lower mappability thresholds were linked with alternative sites. Many of the annotations linked called variants together, i.e. into clusters. Relative to the most strict call set, the least strict set contained around 800 000 additional variants that were linked into more than 600 000 variant clusters. This demonstrates that a substantial fraction of the cell line's total genetic variation is hidden in low mappability regions and that a considerable number of FPs can be avoided using the thesaurus annotation. As an aside, we note that the absolute number of novel variants in a diploid human sample or in a clonal mixture would be expected to be higher.

Among the called variants that were annotated with alternate sites, many were located in exons or candidate functional regions as defined by the Encyclopedia of DNA Elements (23,24): pseudo genes, transcription factor binding sites, DNAse hypersensitivity sites or regions associated with histone marks (Figure 3B). This suggest many of the novel sites may have an influence on the cell line's phenotype. In what follows, we focused our attention on variants found in exonic regions.

Like in the synthetic datasets, thesaurus links connected variants together into clusters. The size of these clusters were typically small (Figure 3C). However, annotations also linked variants to many other additional sites that were not in the original call set. Approximately half of the exonic variants were linked with only one additional site; four-fifths were linked with 10 sites or fewer (Figure 3D). This suggests that a majority of the annotated variants and clusters appear in areas of low repeat copy number and so are in principle amenable to experimental followup.

To check whether the alternate sites carried interpretable information, we checked sequencing error rates and allele frequencies (Supplementary Text section 4). The error rate estimates were lower after accounting for alternate sites. We also observed estimates of allele frequency were closer to expectations in a haploid genome. These results were thus consistent, although less pronounced, than in the synthetic datasets.

Finally, we set out to validate thesaurus clustering experimentally. As the number of novel variants was too large for exhaustive validation, we selected only a few candidates for Sanger sequencing. In order to have the chance of amplifying some candidate sites in a specific manner, we focused our attention on sites that were called with an intermediate mapping quality. We further selected candidates that were in exonic regions, that were linked with a higher-confidence variant and that were in a region of non-zero expression as assessed from a separate RNA-seq dataset. Out of seven candidate clusters picked for validation (Supplementary Text section 4), only one failed to reveal a variant at the targeted site. In four cases we were able to infer the true variation site using specific primers (Figure 3E). In the others, we amplified and sequenced two similar regions simultaneously and observed apparently heterozygous Sanger traces from a haploid genome. In five clusters, the Sanger traces presented evidence for additional variants that were nearby. Upon manual inspection, we found these variants were also identified at low, but not at high, mappability thresholds. Some of these variants annotated with thesaurus and linked with their correct locations. Others were not annotated and were identified at their correct loci. Together, these results show that short-read data aided by the thesaurus resource can indeed uncover variation in low-mappability regions.

### Application to a panel of exomes

Following in-depth analysis of one cell line's genome, we applied the thesaurus technique to study a panel of exome samples. We studied 58 samples originating from breast cancer cell lines (25) and 812 samples from healthy human donors from several human populations of the 1000 Genomes Project (26). After alignment and quality control (Supplementary Text section 5), we called variants at two mappability thresholds and annotated them with the thesaurus. We then identified sites called in low mappability regions that were missed using an intermediate mappability threshold, i.e. sites that were not themselves called at intermediate mappability settings and that were not linked to sites called at intermediate mappability settings. To reduce the number of studied sites, we again focused our attention only on coding regions of Gencode genes.

**Figure 4.**
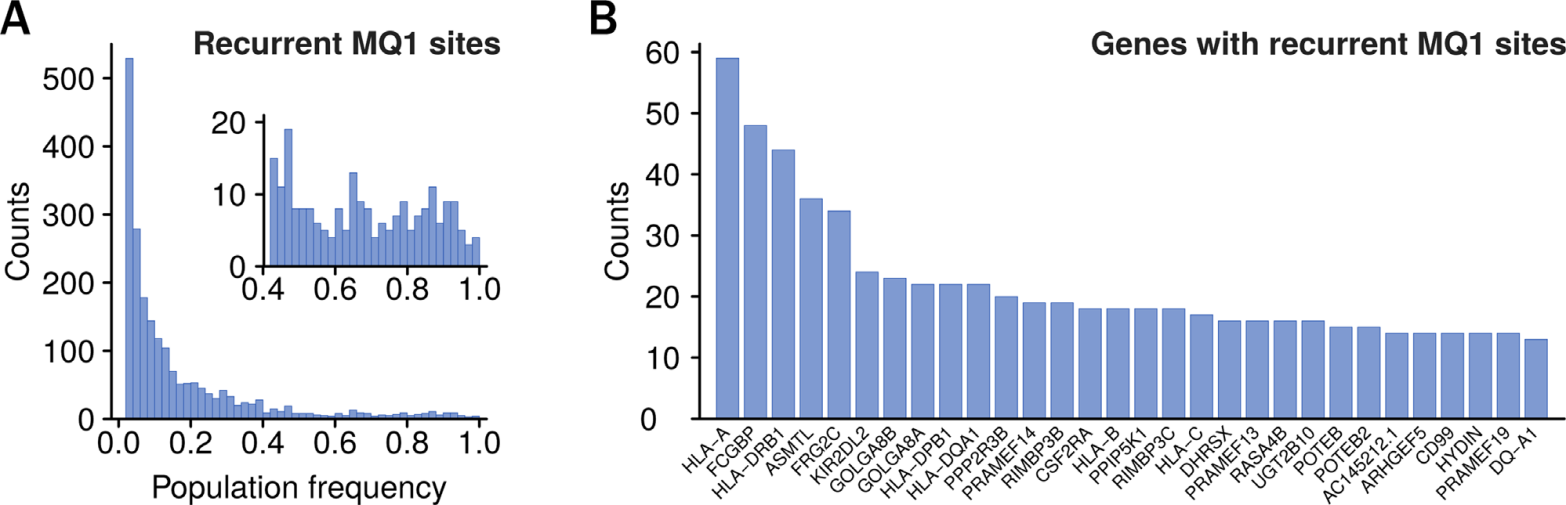
Recurrent variants in low mappability regions of human exomes. (**A**) Histogram of allelic frequencies of variants in a cohort of 872 exome samples. The set includes all variants called at MMQ1 but not at MMQ16, in coding regions of exons and linked via thesaurus annotation to at most two other sites. The inset shows a larger view of the tail of the distribution. (**B**) Ranked list of genes harboring multiple polymorphisms detected at MMQ1 but not at MMQ16.

We searched for sites in low mappability regions that were linked to at most two other sites and that were called recurrently in more than 2% of the cohort. We identified 2096 sites distributed over 503 different genes (Figure 4A). Of these, 751 were associated with positions in dbSNP (27). Although the majority of sites were relatively infrequent in the cohort, we found an allelic abundance >0.2 for 572 sites. These results indicate that variants in low mappability regions can become fixed in populations and that several of these sites are missing in the database of variation. Next, we looked at genes with several recurrently detected sites (Figure 4B). This highlighted a number of genes coding for relatively large proteins. We selected 11 regions from genes ASMTL and RASA4B for Sanger validation in the breast cancer cohort (Supplementary Text Section 5). The regions together contained 22 called variants in eight distinct cell lines. Sanger chromatograms confirmed 18 variants in total, with at least one variants in each of the 11 amplified regions. This suggests that the majority of the other candidates also represent true sites of variation. Within the breast cancer cohort, many of the newly discovered variants likely represent benign polymorphisms, but it is plausible that some may also represent cancer related mutations.

## DISCUSSION

In summary, we developed a method for quick and robust variant detection in low-mappability regions. We showed that whereas variant calls at individual sites can be uncertain, clusters of related sites can carry reliable information. In particular, clusters can give confidence to the presence of variants and also help to better estimate their allelic abundance. We showed that analysis of variant clusters in a human genome can reveal up to hundreds of thousands of elements that have hitherto been cumbersome and impractical to study.

Our variant annotation method uses a resource called a thesaurus of genetic variation. The resource summarizes the repetitive structure of a genome in an unbiased manner, independently of any classifications (transposons, long/short interspersed nuclear elements, Alu elements, etc.) The resource is computationally intensive to generate, but once complete can be efficiently applied to annotate variants. We implemented the annotation method as a step analogous to variant filtering. This means that the thesaurus technique can be incorporated in a straightforward way into existing analysis pipelines. The technique opens analysis of repetitive regions without requiring to re-align or otherwise re-process existing datasets.

As example applications, we studied a whole genome dataset as well as a panel of exome datasets. We demonstrated that variants annotated using the thesaurus can be validated experimentally. Our validation rate (above 80%) was comparable to those reported for variants in high mappability regions (28,29), but may improve in the future with better understanding of remaining artefacts. Many open questions remain about the functions of the newly identified variants. Nonetheless, these examples shows that existing whole-genome and exome sequencing data still hold much unexplored information.

Beyond those very specific examples and applications, the thesaurus technique should be seen as general and versatile. Although we built a thesaurus resource for the human genome, the software can be directly applied to any organism for which an assembled reference genome is available. The technique is also adaptable to simultaneous analysis of matched samples. Further applications may include detection of Mendelian disease-causing variants in parent-child trios and somatic mutations in normal-tumor tissue samples (20,30,31) and haplotyping (32).

## AVAILABILITY

Thesaurus software is available at Sourceforge (http://sourceforge.net/projects/geneticthesaurus/). Links to data files, including vcf used in synthetic dataset and thesaurus resources for human genomes hg19 and hg38 are available on the project's Sourceforge wiki pages.

## SUPPLEMENTARY DATA

Supplementary Data are available at NAR Online.

SUPPLEMENTARY DATA
