# Supplementary material for: A thesaurus of genetic variation for interrogation of repetitive genomic regions
Source: Nucleic Acids Res. 2015 Mar 27;43(10):e68. doi: 10.1093/nar/gkv178 (PMC4446415; doi:10.1093/nar/gkv178)
Supplement: SUPPLEMENTARY DATA [file supp_43_10_e68__index.html]

A thesaurus of genetic variation for interrogation of repetitive genomic regions — SUPPLEMENTARY DATA 

# A thesaurus of genetic variation for interrogation of repetitive genomic regions

## SUPPLEMENTARY DATA

**Files in this Data Supplement:**

- SUPPLEMENTARY DATA
- SUPPLEMENTARY DATA
