# Supplementary material for: A thesaurus of genetic variation for interrogation of repetitive genomic regions
Source: Nucleic Acids Res. 2015 Mar 27;43(10):e68. doi: 10.1093/nar/gkv178 (PMC4446415; doi:10.1093/nar/gkv178)
Supplement: SUPPLEMENTARY DATA [file supp_gkv178_nar-03253-met-n-2014-File007.pdf]

Supplementary Text: A thesaurus of genetic variation for interrogation of repetitive genomic regions

C. Kerzendorfer[1,*], T. Konopka[1,2,*], and S.M.B. Nijman[1,2,*]

[1] *Research Center for Molecular Medicine of the Austrian Academy of Sciences (CeMM), Vienna, Austria*

[2] *Present address: Ludwig Institute for Cancer Research, University of Oxford, Oxford, UK*

[*] *Authors in alphabetical order*

Contents

1 Supplementary Methods **2**
- 1.1 Thesaurus generation . . . 2
- 1.2 Alignments . . . 2
- 1.3 Running times . . . 2
- 1.4 Sanger validation . . . 2

2 Results - single-end synthetic dataset **3**
- 2.1 Independence of local calling approach . . . 3
- 2.2 Thesaurus annotation . . . 4
- 2.3 Properties of annotated variants . . . 6

3 Results - paired-end synthetic dataset **7**
- 3.1 Thesaurus calls . . . 7
- 3.2 Properties of annotated variants . . . 8

4 Results - KBM7 cell line **9**
- 4.1 Properties of annotated variants . . . 9
- 4.2 Sanger Validation in KBM7 . . . 10
- 4.3 Functional mapping . . . 11

5 Results - Exome cohorts **12**
- 5.1 Sample processing . . . 12
- 5.2 Variant analysis . . . 14
- 5.3 Sanger validation in breast cancer cell lines . . . 15

References **17**

1 Supplementary Methods

We collect here supplementary notes on the software and settings used throughout the analysis.

1.1 Thesaurus generation

Thesaurus generation was performed in two main stages. First, we created synthetic reads from the reference genome and created files with exhaustive multiple mappings for each read. There alignments were executed on a compute cluster (approximate running time: 8000 processor hours). Second, we compiled the information in the multiple alignments into a single thesaurus table for the entire genome. These post-alignment computations were performed on a four-core server (approximate running time: 100 processor hours). All steps of the procedure were implemented in custom software written in Java using Picard (http://sourceforge.net/projects/picard) and other third-party libraries.

1.2 Alignments

We aligned synthetic and KBM7 datasets onto the hg19 reference genome using Bowtie2 (v2.0.8) [1] using the predefined `--very-sensitive` settings. In calculations using an alternate fast aligner and for the exome planel, we used GSNAP (v2013-07-30 and v201407-04) [2] together with an index created with settings `-k 14 -q 1`.

1.3 Running times

The approximate running time for variant calling on a 30x whole genome dataset with Bamformatics was around 2 hours. The running time for exomes was around 30 minutes.

The approximate running time for thesaurus filtering of a call set based on a 30x whole-genome dataset was around 8 hours (single processor with access to 24 GB of memory). Lower coverage datasets ran faster and required less memory. Analysis of one exome dataset was around 30 minutes.

1.4 Sanger validation

For Sanger validation, we cultured cells as described by the cell providers, extracted DNA using a Qiagen DNA-extraction kit, and amplified regions of interest via PCR (see below for primer sequences). Sanger sequencing was performed by MicroSynth GmbH, Vienna, Austria.

2 Results - single-end synthetic dataset

The single-end synthetic dataset (described in the main text) consists of reads sampled at regular intervals from a haploid genome based on hg19, but containing a set of randomly placed substitution variants. Alignments were generated with Bowtie 2 [1].

2.1 Independence of local calling approach

We called variants with three distinct variant callers, GATK v.3.2.2 [3], Varscan v2.3.7 [4], and Bamformatics v0.1.2/3 (`http://sourceforge.net/projects/bamformatics/`). We aimed to explore the effect of mappability on each of these callers.

- For GATK, we produced one set of cals with the `UnifiedGenotyper` tool with defaults settings. From this single call set, we produced subsets by filtering by the `MQ` tag associated with each called variant. We also tried to use the `Haplotypecaller`, but we noted this produced fewer variants than the UnifiedGenotyper on our datasets.
- Varscan does not operate directly on alignment files, but rather uses input from `samtools mpileup`. To vary mapping quality thresholds, we thus repeated the calling procedure for each mapping quality threshold and changed the `-q` argument when running `samtools mpileup`. All other Varscan settings were left at the default values.
- For Bamformatics, we called varaints independently for each mapping quality threshold by varying the `--minmapqual` setting. All other settings were left at the default values.

Although the interpretation of the threshold varies across the methods (Table S1), ROC analysis shows that the overall performance of the three callers is comparable (Figure S1). This is not surprising because the synthetic dataset did not contain difficult features other than variants in low mappability regions. Since all three callers process the data one position/region at a time and produce output in vcf format, they all encounter the same limitations in repetitive regions.

	GATK			Varscan			Bamformatics		
MQ	TP	FP	FN	TP	FP	FN	TP	FP	FN
0	2734774	32	161992	2777987	15841	118779	2830013	87071	66753
1	2734774	32	161992	2777965	15831	118801	2830030	86241	66736
2	2734774	32	161992	2727978	33	168788	2778074	399	118692
3	2734774	32	161992	2727947	33	168819	2778069	300	118697
4	2734774	32	161992	2727939	32	168827	2778069	294	118697
6	2734774	32	161992	2727926	32	168840	2778068	285	118698
8	2734758	31	162008	2692080	3	204686	2750550	88	146216
10	2733965	20	162801	2692080	3	204686	2750550	88	146216
12	2729948	16	166818	2689488	3	207278	2749132	80	147634
16	2707969	10	188797	2665764	1	231002	2729225	28	167541
20	2679023	2	217743	2622416	1	274350	2693874	5	202892
24	2639792	2	256974	2601299	1	295467	2685312	3	211454
28	2574743	1	322023	2390899	0	505867	2558957	2	337809
32	2508043	1	388723	2390899	0	505867	2558957	2	337809
36	2430046	0	466720	2347200	0	549566	2506832	2	389934
40	2301667	0	595099	2347200	0	549566	2506832	2	389934

Table S1: **Variant calling performance with three local variant callers.** Columns marked GATK, Varscan, and Bamformatics represent results obtained from three distinct variant callers. MQ: mapping quality threshold. TP, FP, FN: absolute numbers of true positives, false positives, and false negatives.

2.2 Thesaurus annotation

We explored the performance gain due to thesaurus annotation. We decided to use calls from Bamformatics because this caller produced the largest call set at the low mappability thresholds; it thus produced the most candidates that could in principle represent true hits. We performed thesaurus filtering using default settings, but with minimum mapping quality set to match that used during variant calling. A comparison with true calls appears in Table S2.

We also ran control calculations to make sure that the performance gains are not due to spurious connections between genomic positions. We generated files with random links between thesaurus-annotated variants and other genomic positions in low mappability regions, following the distributions of the true thesaurus links (i.e. if a site was linked with three other sites, we generated three random links). We found random links did not give rise to substantial performance improvements (Table S2).

For ROC analysis, we used the following definitions of “false positive rate” (FPR) and “thesaurus true positive rate” (TTPR):

$$[FPR] = \frac{[FP]}{[FP] + [TN]}, \tag{1}$$

$$[TTPR] = \frac{[TP] + [TTP]}{[TP] + [TTP] + [FN]}, \tag{2}$$

where we used $[TN] = 3 \times 10^9$ for the human genome. A ROC-style representation of the results appears in Figure S1.

We also ran thesaurus annotation on select sets of calls from the other variant callers. In all cases, the results were qualitatively similar to those found based on Bamformatics (data not shown).

	Bamformatics				with Thesaurus				with Random			
MQ	TP	FP	FN	TTP	TP	FP	FN	TTP	TP	FP	FN	TTP
0	2830013	87071	66753	0	2829609	143	30448	85343	2830013	86558	66660	490
1	2830030	86241	66736	0	2829627	52	30441	85304	2830030	85754	66648	482
2	2778074	399	118692	0	2777946	26	118540	227	2778074	379	118687	15
3	2778069	300	118697	0	2777943	23	118545	225	2778069	286	118692	11
4	2778069	294	118697	0	2777943	20	118545	224	2778069	280	118692	11
6	2778068	285	118698	0	2777942	19	118546	222	2778068	271	118694	12
8	2750550	88	146216	0	2750487	8	146158	67	2750550	81	146213	7
10	2750550	88	146216	0	2750487	8	146158	67	2750550	81	146213	7
12	2749132	80	147634	0	2749069	6	147581	61	2749132	72	147631	8
16	2729225	28	167541	0	2729196	1	167524	19	2729225	25	167538	3
20	2693874	5	202892	0	2693866	0	202888	5	2693874	5	202892	0
24	2685312	3	211454	0	2685306	0	211451	3	2685312	3	211454	0
28	2558957	2	337809	0	2558957	0	337807	2	2558957	2	337809	0
32	2558957	2	337809	0	2558957	0	337807	2	2558957	2	337809	0
36	2506832	2	389934	0	2506832	0	389932	2	2506832	2	389934	0
40	2506832	2	389934	0	2506832	0	389932	2	2506832	2	389934	0

Table S2: **Thesaurus performance on synthetic dataset.** Group labeled “Bamformatics” shows performance of local variant calling using Bamformatics (v0.1.2/v0.1.3). Group ”with Thesaurus” gives performance of Bamformatics calls augmented by thesaurus annotations. Group “with Random” gives performance of Bamformatics calls augmented by randomly generated links mimicking thesaurus annotation. MQ: minimum mapping quality used during variant calling. TP, FP, FN, and TTP: absolute numbers of true positives, false positives, false negatives, and thesaurus true positives. Total number of variants under “Bamformatics” and “with Thesaurus” differ because variants labeled as `thesaurushard` or `thesaurusmany` were eliminated from the analysis in the latter case.

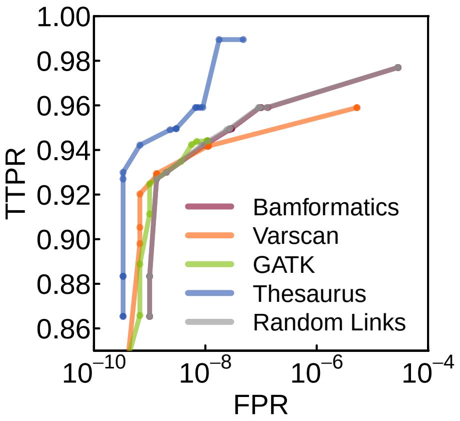

Figure S1: **ROC-style representation of calling performance using three variant callers.** ROC-style curves show performance of traditional and thesaurus-aided variant calling. Red, orange, and green lines represent performnace of three local variants callers: Bamformatics, Varscan, and GATK, respectively. Blue curve shows performance of Thesaurus annotation based on Bamformatics output. Gray curve, which overlaps the curve, shows a lack of performance change for Bamformatics output augmented by randomly generated links mimicking thesaurus annotation.

2.3 Properties of annotated variants

We explored various properties of the annotated variants and the alternate variant loci (Figure S2). When using a very low mapping quality threshold (MQ 1), several of the called variants could be connected together via thesaurus links. The size of these clusters (only among the called variants) was rarely greater than three (Figure S2A). However, the number of alternate loci associated with called sites ranged from one to many thousand, with the majority of sites linking to ten or fewer (Figure S2B).

We then calculated the B-allele frequencies (BAF) for variants annotated with the thesaurus resource. When we estimated the frequency using only the reads at the called position, we found most sites had BAFs substantially smaller than one, which is incorrect since the synthetic genome was haploid. Alternate loci were valuable in better estimating the BAF for these variants (Figure S2C)

We also noted that accounting for alternate loci substantially decreased the apparent error rate on non-called sites (Figure S2D). To evaluate error rates, we used the `errors` tool in the Bamformatics toolkit with parameters set to `--maxallelic 0.05 --maxdepth 3`. We restricted attention to specific regions and noted that the apparent error rate was three orders of magnitude lower in well-mappable areas than in non-mappable areas. The error estimate was much reduced after removing alternate sites found during filtering, but not when removing the same number of randomly picked loci within low-mappability regions (Figure S2D). The error rate did not drop to zero after accounting for alternate sites, possibly because some variants still remained undiscovered (see ROC curve, and FN in Table S2).

When using higher mapping quality thresholds (e.g. MQ 16 in the figure), the number of sites annotated by thesaurus filtering was much reduced, and consequently the effects were less pronounced. Interestingly, the error rate among reads with high mapping quality was still two orders of magnitude higher on thesaurus covered regions than on the rest of the genome. This suggests that some reads assigned with high MQ during alignment are actually misplaced.

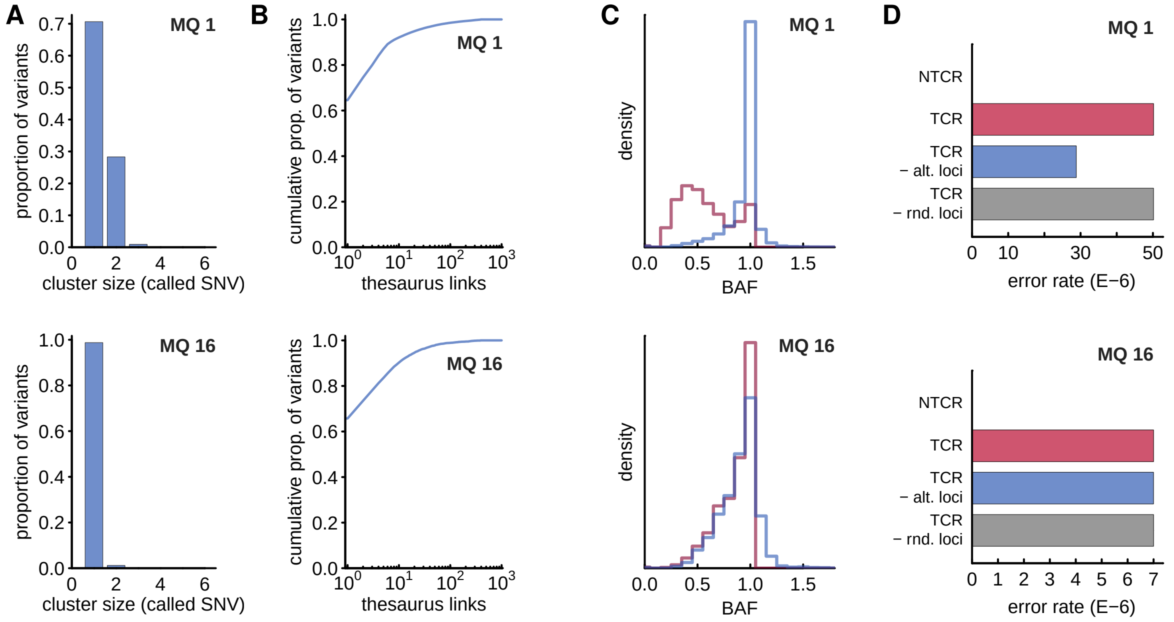

Figure S2: **Properties of thesaurus annotations on synthetic data (single-end). (Top row)** Results obtained with mapping quality (MQ) threshold 1. **(Bottom row)** Results with MQ threshold 16. **(A)** Cluster sizes among the variants annotated with the thesaurus (if a called variant is annotated with several alternate loci, of which only one is a called variant, the cluster size is two) **(B)** Cumulative distribution function of thesaurus links associated with annotated variants. **(C)** Distribution of B-allele frequencies attributed to thesaurus-annotated variants: red shows local estimates based on read counting, blue shows estimates based on read counting using alternate loci. **(D)** Error rates on genomic regions. (NTCR) rate on regions not covered by the thesaurus resource. (TCR) rate on thesaurus-covered regions. (TCR-alt.loci) rate on TCR regions avoiding sites identified during thesaurus filtering. (NTCR-rnd.loci) rate on low-mappability regions avoiding randomly selected sites (repeated five times, error bars are too small to see).

3 Results - paired-end synthetic dataset

Following tests with single-end data, we repeated the analysis on analogous paired-end data based on the same genome. We generated paired-end pairs at regular intervals of 20bp and with insert size of 290; overall this scheme gives data with expected coverage of 10× as before. Alignments were again generated with Bowtie 2.

3.1 Thesaurus calls

As for the single-end datasets, we generated initial calls using GATK, Varscan, and Bamformatics and found the three callers to be comparable (Figure S3). We thus again chose to work with calls from Bamformatics. Thesaurus filtering was performed using default settings, but with minimum mapping quality set to match those used for variant calling (Table S3 and Figure S3). In comparison with the single-end dataset, the number of calls was larger because paired-end reads were effectively longer (200bp vs 100bp) and could thus be mapped with more confidence. Regardless, thesaurus filtering still dramatically improved performance, bringing the (thesaurus) true positive rate very near to unity (Figure S3).

	Bamformatics				with Thesaurus				with Random			
MQ	TP	FP	FN	TTP	TP	FP	FN	TTP	TP	FP	FN	TTP
0	2865191	71406	31575	0	2852539	12	8616	71004	2865191	71104	31558	302
1	2865194	71406	31572	0	2852539	12	8613	71004	2865194	71105	31549	301
2	2819633	57	77133	0	2807510	0	77115	26	2819633	54	77133	3
3	2819633	57	77133	0	2807510	0	77115	26	2819633	54	77133	3
4	2819633	57	77133	0	2807510	0	77115	26	2819633	54	77133	3
6	2819633	57	77133	0	2807510	0	77115	26	2819633	54	77133	3
8	2782820	7	113946	0	2774295	0	113946	0	2782820	7	113946	0
10	2782820	7	113946	0	2774295	0	113946	0	2782820	7	113946	0
12	2782673	7	114093	0	2774169	0	114093	0	2782673	7	114093	0
16	2757133	7	139633	0	2751794	0	139633	0	2757133	7	139633	0
20	2716571	7	180195	0	2714712	0	180195	0	2716571	7	180195	0
24	2697043	1	199723	0	2695569	0	199723	0	2697043	1	199723	0
28	2645923	1	250843	0	2644535	0	250843	0	2645923	1	250843	0
32	2645923	1	250843	0	2644535	0	250843	0	2645923	1	250843	0
36	2641167	1	255599	0	2639778	0	255599	0	2641167	1	255599	0
40	2641167	1	255599	0	2639778	0	255599	0	2641167	1	255599	0

Table S3: **Thesaurus performance on synthetic dataset - paired-end.** Analogous to Table S2, but based on paired-end data.

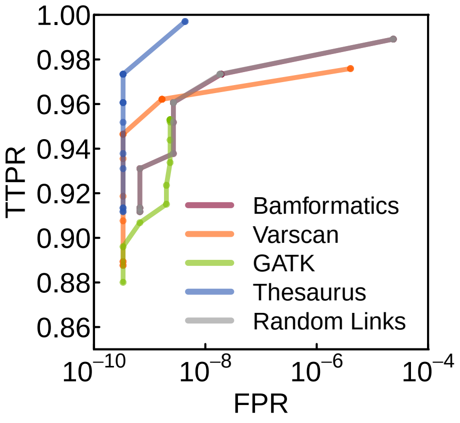

Figure S3: **ROC-style representation of calling performance using three variant callers - paired-end.** Analogous to Figure S1, but based on paired-end data.

3.2 Properties of annotated variants

We explored various properties of the annotated variants and the alternate variant loci analogous as for the single-end dataset (Figure S4). Overall, the results were qualitatively similar to those in the single-end dataset. Clusters typically involved three called sites or fewer. Annotations linked called sites to many other sites, sometimes hundreds of sites. BAF estimates were more appropriate using alternative sites. Error estimates were lower when accounting for sites linked to called variants. Notably, the error estimates decreased further here than in the single-end dataset, possibly because there were fewer undetected variants overall.

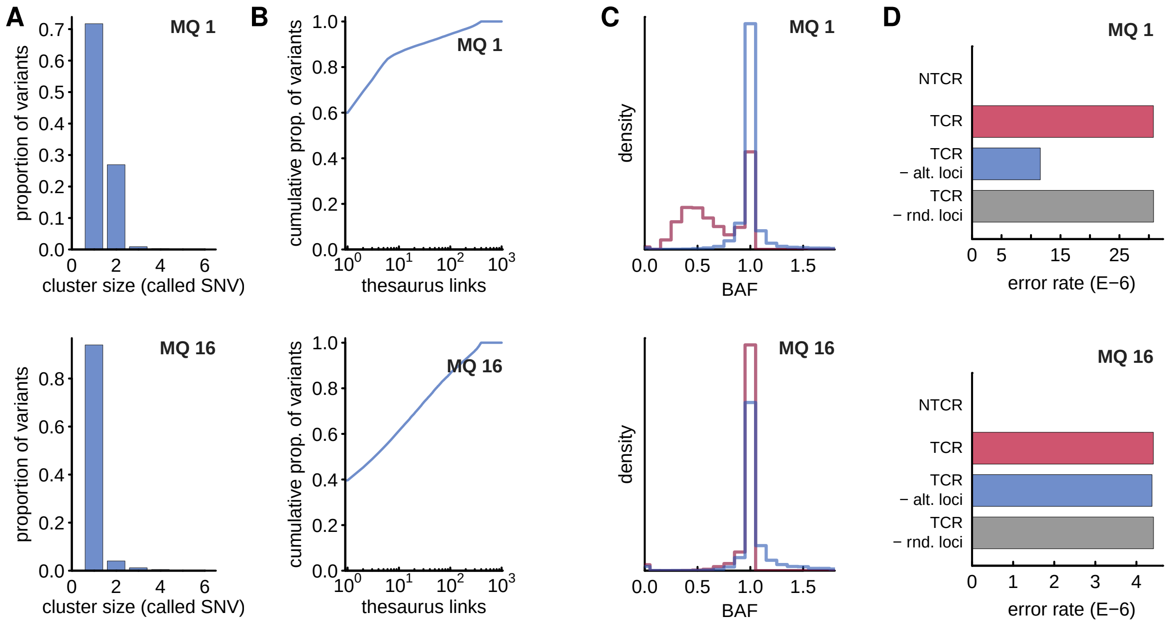

Figure S4: **Properties of thesaurus annotations on synthetic data (paired-end)** Analogous to Figure S2, but here using paired-end synthetic data. (Top row) data obtained from variants detected with low mapping quality threshold (MQ1). (Bottom row) data obtained from variants called at high mapping quality threshold (MQ16).

4 Results - KBM7 cell line

We studied thesaurus annotation in a real whole-genome data from the KBM7 cell line. The KBM7 cell line is diploid on chromosome 8 and a portion of chromosome 15, but is otherwise haploid. We used a whole-genome dataset with 2x100bp reads with $\sim 30\times$ median coverage.

4.1 Properties of annotated variants

We evaluated properties of thesaurus-annotated variants in the KBM7 cell line in a similar fashion as for synthetic data (Figure S5).

Compared to the synthetic dataset, the size of clusters formed between called variants was slightly higher (Figure S5A) and the number of alternate loci, despite filtering based on paired-end reads, was sometimes substantially higher (Figure S5B). This may reflect local copy-number variation of low-mappability regions, for example in transposable elements, centromeres, and telomeres.

Alternate sites affected the BAF profile of annotated sites (Figure S5C). Thesaurus-correction removed very low frequencies. However, it did not shift the overall distribution to one perfectly peaked around a single value. This could be due to a number of factors: short-range copy number variation in the cell line may not be reflected in the reference sequence, variants linking to diploid regions may be polluting the results, or depth differences due to GC content or other factors may need to be incorporated into the analysis.

As all real data, the KBM7 reads contained random and systematic sequencing errors in addition to mismatches due to misalignment. Thus, the measured error rate was non-negligible throughout the genome (Figure S5D). Still it was substantially higher in regions of low mappability. Removing alternate sites reduced the estimated error rate (significantly, as compared to removing sets of randomly selected sites, but slightly, as evaluated by a fold change), but not as dramatically as in the synthetic dataset. This suggests that a large number of variants are still undiscovered, or that the documentation of alternate loci was too strict.

At high mapping quality thresholds, the number of identified clusters and cluster sizes decreased. The estimated error rates were equivalent on thesaurus-covered and non-thesaurus-covered regions, indicating that sequencing errors were a larger hindrance in variant detection than misalignments.

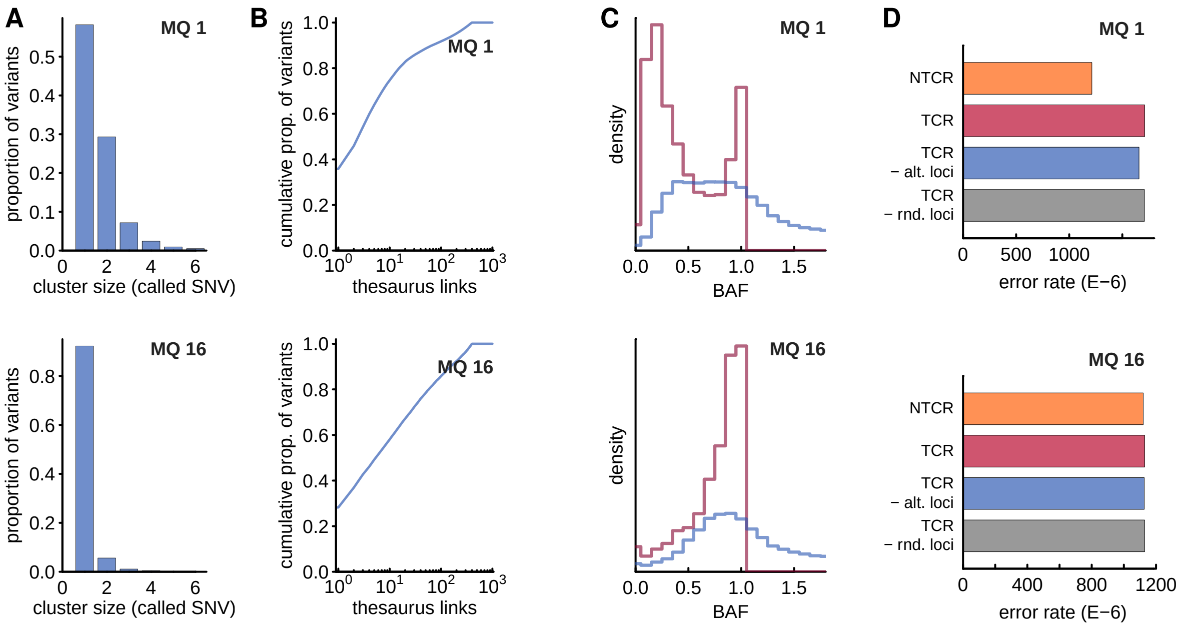

Figure S5: **Properties of thesaurus annotations on KBM7 data.** Analogous to Figure S2 but using whole-genome data from the KBM7 cell line.

4.2 Sanger Validation in KBM7

Sanger sequencing was performed on seven clusters of called variants. Primers used are shown in Table (S4). Sanger traces (forward and reverse sequencing) are provided as Supplementary data file (`KBM7.Thesaurus.Sanger.zip`).

Cell Line	Site	Gene	Primer Fw	Primer Rev
KBM7	chr1:1666251<br>chr1:1599888	SLC35E2<br>SLC35E8	CTCCTTTGTAAACAACCTGG	GGATGAGTGCACAGTTCAGC
KBM7	chr7:151945101<br>chr21:11038843	MLL3<br>BAGE2	CCCTACCTGTTTGGACCGAG	GTGTCCCCACATGAGGAAAG
KBM7	chr7:151945225<br>chr21:11038967	MLL3<br>BAGE2	GTCATGCGAAGGCAAGTCTG	ACACCAGATCACTGTGCAGC
~~KBM7~~	~~chr7:151962168~~<br>~~chr21:11049495~~	~~MLL3~~<br>~~BAGE2~~	CTTACTTGCAGTTCTGGCAC	CGGGAGACCTCTTAGATCAG
KBM7	chr16:1279438<br>chr16:1291454<br>chr16:1306817	TPSB2<br>TPSAB1<br>TPSD1	CATTGTCCACATCGCCCCAG<br>GTGAGCCTGAGAGTCCACGG<br>CAGCAAACGGGCATTGTTGG	AGCCTGAGAGTCCGCGACCG<br>TAAGACCCTGGCCCCACCTC<br>TGGCTGGAGATGTTCACGGG
KBM7	chr17:33769034<br>chr17:33680807	SLFN13<br>SLFN11	CAAGGCTATAGGACGCAGGG<br>CAAGGCTATAGGACGCAGGG	TCCTGTCTAGTTCCACCAGG<br>TTCACATACAGTCCCACCAG
KBM7	chr19:33490566<br>chr15:20453992	RHPN2<br>intergenic	CAAAGTGGCCACGATGACTC<br>TCTGAGGCTCAGGACTGCAC	TCCGGGGGCAGAAAGGAGAC<br>TGGCCAGATCCATGAGAGGG

Table S4: **Sanger validation.** Blocks of lines indicate clusters of variants linked via the Thesaurus. Where one set of primers is shown, the product was expected to consist of a mixture of both targeted sites. Where multiple primers are shown, each pair was expected to preferentially amplify one of the targeted sites in the cluster. Crossed out entries represent experiments that yielded Sanger traces, but did not show the called variants.

All reactions except those targeting MLL3:chr7:151962168 demonstrated presence of variants at the called positions. In cases where primers were unspecific, Sanger traces showed evidence of multiple alleles (Figure S6). While this is evidence of heterozygous variants in well-mappable regions, this is here indicative of amplification of multiple genomic regions (KBM7 is haploid on the targeted chromosomes).

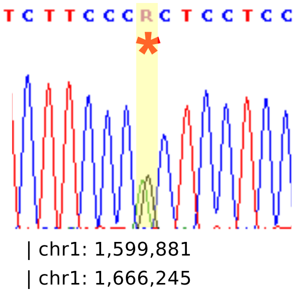

Figure S6: **Sanger validation of low-mapping quality variant in KBM7.** The variant is present on SLC35E2/SLC35E8. The relative proportion of the two alleles is consistent with one variant on a haploid chromosome.

Specific primers were able to distinguish the true site of variation from misalignments. Since the candidate sites were selected with one locus called at higher mapping quality, the higher-mapping-quality variant was always the true site of variation. However, the ploidy of the variants differed in the calls produced by NGS and seen in the Sanger traces (Figure main text).

4.3 Functional mapping

To start to assess the importance of the newly called variants, we compared the called loci with functional annotation tracks from ENCODE [5] (Table S5). Although the annotation data are individually available through the UCSC genome browser, we compiled the `bed` files used in the calculation in supplementary data (`Thesaurus.ENCODE.zip`).

Label	Description
Exon	Exonic regions, Gencode annotation set (V19)
Exon.1K	Exonic regions and 1kb flanking regions, Gencode annotation set (V19)
Pseudo	Pseudo genes, Gencode annotation set (V19)
TF	Transcription factor binding sites, merge of 161 binding factors on several cell lines (ENCODE-prepared download)
DNase	DNase hypersensitivity regions, merge of cell types (ENCODE-prepared download)
H3K4me1	Histone methylation regions, produced manually by merging data from 7 cell lines
H3K4me3	Histone methylation regions, produced manually by merging data from 7 cell lines.
H3K27Ac	Histone acetylation regions, produced manually by merging data from 7 cell lines.

Table S5: **ENCODE annotations used for functional mapping.** Label refers to the name of the track used in the main text. Description is a short explanation of the track. Histone tracks are manual pools obtained by superposing tracks from the following cell lines: GM12878, H1-hESC, HSMM, HUVEC, K562, NHLEK, and NHLF.

5 Results - Exome cohorts

5.1 Sample processing

We processed exome samples from breast cancer cell lines [6] and healthy human individuals from around the world [7]. Based on metadata from the SRA (`http://www.ncbi.nlm.nih.gov/sra`), we selected samples marked "WXS" (whole exome sequencing) containing runs of moderate size. We then downloaded unaligned data and applied the following data-processing pipeline. We removed reads with low-base-quality and identical sequences using Triagetools [8]. We aligned the remaining data onto the hg19 reference genome using GSNAP [2].

In the breast cancer cohort, we noticed that many paired reads had very short insert sizes, often leading overlapping paired reads to run into adapter sequences. Although GSNAP typically clips such artifacts, we nonetheless noticed several calls that seemed artefactual due to such reads. In this cohort only, we therefore identified reads that contained more than clipped 10 bases and removed the from the alignments.

The samples in the cell line cohort and the 1000 genomes cohorts were not all sequenced following the same protocol (read length, insert sizes, etc.) In order to reduce the influence of readlength on mappability, we identified samples containing reads shorter than 95 bases and excluded them from the remaining analysis. We also excluded data labeled MDAMB175VII, as we noticed this data to be a duplicate of data for cell line MDAMB157. The remaining 871 samples are shown in Table S6.

**Cohort**	**Samples**					
BCCL	184A1	184B5	21MT1	21MT2	21NT	21PT
(SRP026538)	600MPE	AU565	BT20	BT474	BT483	BT549
	CAL120	CAL148	CAL51	CAL851	CAMA1	EFM19
	EFM192A	EFM192B	EFM192C	EVSAT	HCC1143	HCC1143BL
	HCC1187	HCC1395	HCC1428	HCC1937	HCC1954	HCC2185
	HCC2218	HCC38BL	HDQP1	JIMT1	LY2	MCF10A
	MCF10F	MCF12A	MDAMB134VI	MDAMB157	~~MDAMB175VII~~	MDAMB361
	MFM223	MT3	MX1	PMC42	SKBR3	SUM102PT
	SUM185PE	SUM225CWN	SUM229PE	SUM44PE	SUM52PE	T4
	T47D_KBluc	UACC893	ZR751	ZR7530	ZR75B	
ACB	HG01886	HG01889	HG01890	HG01894	HG01912	HG02009
(SRP004069)	HG02010	HG02014	HG02054	HG02255	HG02256	HG02281
	HG02282	HG02317	HG02318	HG02449	HG02450	HG02470
	HG02471	HG02476	HG02477	HG02478	HG02479	HG02481
	HG02484	HG02485	HG02489	HG02496	HG02497	HG02501
	HG02502	HG02505	HG02508	HG02511	HG02536	HG02537
	HG02545	HG02546	HG02557	HG02558	HG02577	
ASW	NA19922	NA19923	NA19984	NA19985	NA20126	NA20127
(SRP004055)	NA20276	NA20278	NA20281	NA20282	NA20287	NA20289
	NA20291	NA20294	NA20296	NA20298	NA20299	NA20313
	NA20314	NA20317	NA20322	NA20332	NA20334	NA20336
	NA20339	NA20340	NA20341	NA20342	NA20344	NA20346
	NA20348	NA20351	NA20356	NA20357	NA20359	NA20361
	NA20362	NA20412				
CDX	HG02152	HG02153	HG02154	HG02155	HG02156	HG02164
(SRP004062)	HG02165	HG02166	HG02168	HG02169	HG02170	HG02173
	HG02176	HG02178	HG02179	HG02180	HG02181	HG02182
	HG02184	HG02185	HG02186	HG02187	HG02188	HG02190
	HG02358	HG02405				
CEU	GM06985	GM07000	GM07056	GM07357	GM10847	GM10851
(SRP004078)	GM11829	GM11831	GM11832	GM11840	GM11992	GM11993
	GM11994	GM12003	GM12004	GM12005	GM12006	GM12043
	GM12046	GM12154	GM12156	GM12249	GM12273	GM12275
	GM12283	GM12414	GM12716	GM12718	GM12751	GM12761
	GM12763	GM12813	GM12814	GM12872	GM12873	GM12874
	NA06994	NA11830	NA11881	NA11995	NA12044	NA12144
	NA12155	NA12234	NA12272	NA12282	NA12286	NA12489
	NA12717	NA12760	NA12762	NA12812	NA12815	
CHB	NA18525	NA18527	NA18528	NA18531	NA18591	NA18614
(SRP004364)	NA18615	NA18617	NA18618	NA18619	NA18625	NA18626
	NA18627	NA18628	NA18629	NA18630	NA18639	NA18640
	NA18641	NA18642	NA18643	NA18644	NA18645	NA18646
	NA18647	NA18648	NA18740	NA18745	NA18747	NA18748
	NA18749	NA18757	NA18791	NA18794	NA18795	
CHS	HG00559	HG00560	HG00565	HG00566	HG00592	HG00593
(SRP004365)	HG00595	HG00596	HG00716	HG00717		

CLM	HG01112	HG01113	HG01124	HG01125	HG01133	HG01134
(SRP004070)	HG01136	HG01137	HG01139	HG01140	HG01148	HG01149
	HG01250	HG01251	HG01253	HG01254	HG01271	HG01272
	HG01274	HG01275	HG01277	HG01278	HG01344	HG01345
	HG01347	HG01348	HG01356	HG01357	HG01360	HG01452
	HG01453	HG01455	HG01456	HG01468	HG01471	HG01473
	HG01474	HG01477	HG01479	HG01480	HG01482	HG01483
	HG01494	HG01495	HG01497	HG01498	HG01550	HG01551
FIN	HG00173	HG00177	HG00178	HG00179	HG00180	HG00181
(SRP004058)	HG00182	HG00183	HG00185	HG00186	HG00187	HG00188
	HG00189	HG00190	HG00266	HG00267	HG00269	HG00270
	HG00302	HG00303	HG00304	HG00349	HG00350	HG00351
	HG00355	HG00356	HG00358	HG00359	HG00360	HG00362
	HG00364	HG00365				
GBR	HG00096	HG00097	HG00099	HG00101	HG00102	HG00103
(SRP004060)	HG00104	HG00105	HG00106	HG00107	HG00112	HG00114
	HG00115	HG00118	HG00122	HG00126	HG00127	HG00128
	HG00129	HG00130	HG00132	HG00134	HG00135	HG00141
	HG00142	HG00143	HG00148	HG00149	HG00150	HG00151
	HG00152	HG00156	HG00231	HG00233	HG00234	HG00235
	HG00238	HG00240	HG00247	HG01789	HG01790	HG04301
	HG04302	HG04303				
GIH	NA20882	NA20883	NA20884	NA21089	NA21090	NA21091
(SRP004056)	NA21092	NA21094	NA21097	NA21098	NA21099	NA21100
	NA21101	NA21102	NA21103	NA21104	NA21105	NA21106
	NA21107	NA21108	NA21109	NA21110	NA21111	NA21112
	NA21113	NA21115	NA21116	NA21117	NA21118	NA21119
	NA21120	NA21121	NA21122	NA21123	NA21125	
GWD	HG02762	HG02763	HG02869	HG02870	HG03033	HG03034
(SRP004065)	HG03249	HG03250				
IBS	HG01500	HG01501	HG01503	HG01504	HG01515	HG01516
(SRP004061)	HG01518	HG01519	HG01521	HG01522	HG01602	HG01603
	HG01605	HG01606	HG01607	HG01608	HG01610	HG01612
	HG01613	HG01615	HG01617	HG01618	HG01619	HG01620
	HG01623	HG01624	HG01625	HG01626	HG01628	HG01630
	HG01668	HG01669	HG01670	HG01672	HG01756	HG01757
	HG01761	HG01762	HG01770	HG01771	HG01773	HG01775
	HG01776	HG01777	HG01779	HG01781	HG01783	HG01784
	HG01785	HG01786	HG02218	HG02219	HG02220	HG02221
	HG02235	HG02236				
JPT	NA18939	NA18941	NA18946	NA18954	NA18955	NA18956
(SRP004076)	NA18957	NA18962	NA18963	NA18965	NA18966	NA18967
	NA18968	NA18969	NA18970	NA18971	NA18972	NA18973
	NA18974	NA18975	NA18976	NA18977	NA18978	NA18979
	NA18980	NA18981	NA18987	NA18990	NA18991	NA18992
	NA18993	NA18994	NA18995	NA18997	NA18998	NA19001
	NA19002	NA19005	NA19009	NA19010	NA19072	NA19074
	NA19075	NA19076	NA19077	NA19079	NA19080	NA19081
	NA19082	NA19083	NA19084	NA19085	NA19086	
KHV	HG01870	HG01871	HG01872	HG01873	HG01874	HG01878
(SRP004063)	HG02016	HG02017	HG02019	HG02020	HG02035	HG02048
	HG02049	HG02057	HG02058	HG02069	HG02070	HG02072
	HG02073	HG02075	HG02076	HG02078	HG02079	HG02081
	HG02082	HG02084	HG02085	HG02086	HG02087	HG02088
	HG02113	HG02121	HG02122	HG02127	HG02128	HG02138
	HG02139	HG02140	HG02141	HG02524	HG02525	
LWK	NA19020	NA19027	NA19028	NA19031	NA19035	NA19036
(SRP004075)	NA19038	NA19041	NA19044	NA19307	NA19308	NA19309
	NA19310	NA19311	NA19312	NA19313	NA19314	NA19315
	NA19316	NA19317	NA19318	NA19319	NA19321	NA19324
	NA19327	NA19328	NA19331	NA19351	NA19355	NA19359
	NA19360	NA19371	NA19372	NA19374	NA19375	NA19376
	NA19377	NA19379	NA19380	NA19381	NA19383	NA19384
	NA19385	NA19390	NA19391	NA19393	NA19394	NA19395
	NA19397	NA19398	NA19399	NA19401	NA19403	NA19404
	NA19428	NA19429	NA19430	NA19431	NA19434	NA19435
	NA19436	NA19437	NA19438	NA19439	NA19440	NA19443
	NA19445	NA19446	NA19448	NA19449	NA19451	NA19452
	NA19455	NA19456	NA19457	NA19461	NA19462	NA19463
	NA19466	NA19467	NA19468			
MXL	NA19728	NA19729	NA19731	NA19732	NA19734	NA19735
(SRP004054)	NA19737	NA19740	NA19741	NA19746	NA19747	NA19750

	NA19752	NA19755	NA19756	NA19758	NA19759	NA19761
	NA19762	NA19764	NA19770	NA19771	NA19773	NA19774
	NA19776	NA19777	NA19779	NA19780	NA19782	NA19783
	NA19785	NA19786	NA19788	NA19789	NA19794	NA19795
	NA19797	NA19798				
PEL (SRP004071)	HG01565	HG01566	HG01571	HG01572	HG01953	HG01954
	HG01961	HG01965	HG01967	HG01968	HG01970	HG01971
	HG01973	HG01974	HG01976	HG01977	HG01979	HG01980
	HG01991	HG01992	HG01995	HG01997	HG02002	HG02003
	HG02008	HG02089	HG02090	HG02104	HG02105	HG02272
	HG02277	HG02278	HG02285	HG02286	HG02288	HG02291
	HG02292	HG02298	HG02299	HG02301	HG02344	HG02345
	HG02347					
PUR (SRP004072)	HG00742	HG00743	HG01058	HG01063	HG01064	HG01077
	HG01088	HG01089	HG01092	HG01161	HG01162	HG01164
	HG01195	HG01200	HG01286	HG01302	HG01303	HG01305
	HG01308	HG01311	HG01312	HG01322	HG01323	HG01325
	HG01326	HG01398				
TSI (SRP004073)	NA20502	NA20503	NA20504	NA20505	NA20506	NA20507
	NA20508	NA20509	NA20510	NA20512	NA20513	NA20514
	NA20515	NA20516	NA20517	NA20518	NA20519	NA20520
	NA20521	NA20522	NA20524	NA20525	NA20526	NA20527
	NA20528	NA20529	NA20530	NA20531	NA20532	NA20533
	NA20534	NA20535	NA20536	NA20537	NA20538	NA20539
	NA20540	NA20541	NA20807	NA20808	NA20809	NA20810
	NA20811	NA20812	NA20813	NA20814	NA20815	NA20816
	NA20818	NA20819	NA20826	NA20827	NA20828	NA20829
	NA20831	NA20832				
YRI (SRP004074)	NA18502	NA18505	NA18507	NA18508	NA18917	NA18923
	NA18924	NA18933	NA18934	NA19095	NA19096	NA19098
	NA19099	NA19113	NA19117	NA19118	NA19119	NA19121
	NA19131	NA19138	NA19141	NA19143	NA19144	NA19146
	NA19149	NA19150	NA19152	NA19153	NA19159	NA19160
	NA19171	NA19175	NA19184	NA19185	NA19197	NA19198
	NA19200	NA19201	NA19204	NA19206	NA19207	NA19209
	NA19210	NA19214	NA19222	NA19223	NA19238	NA19239
	NA19240					

Table S6: **Exome samples processed for cohort analysis.** The first column indicates cohort names. BCCL stands for Breast Cancer Cell Lines. Other acronyms are population codes defined by the 1000 Genomes project. SRP codes are SRA accession numbers. The second column shows names of the samples from each cohort.

5.2 Variant analysis

After inspecting the alignments, we called variants with mapping quality thresholds set at 16 (MMQ16) and 1 (MMQ1) using Bamformatics. We annotated the variants with dbSNP ids (dbSNP138) and with the thesaurus resource. We flagged variants based on strand bias (tag $SF > 12$), simple-repeat regions (repeatMasker simple repeats and 10 flanking bases), number of distinct read start positions (tag $DS < 3$), mean number of mismatches (tag $NM > 6$), and median mapping quality (tag $MM < 16$).

For each cohort, we first read all the variants called at MMQ16. Then, using calls at MMQ1 and their thesaurus annotations, we identified variants that were newly called at the lower mappability setting and that were not linked to any of the MMQ16 sites. Then, we created subtables of variants in exonic and coding regions of Gencode genes. We split the counts into calls obtained with high and low confidence (based on flags for strand bias, indistinct start positions, large number of mismatches). This allowed us to later discard variants that were always associated with unwanted flags, but include flagged variants when more than two-thirds of samples called a variants without any flags. After processing each cohort separately, we merged the tables together.

5.3 Sanger validation in breast cancer cell lines

We performed Sanger sequencing on several variants called in the breast cancer cell lines (Table S7 and Table S8). Sanger traces are provided as a Supplementary data file (`BCCL.Thesaurus.Sanger.zip`). In all cases, primers were not specific to a single genomic location. Rather, they were meant to amplify more than one genomic region. As such, Sanger chromatograms therefore showed evidence of multiple alleles (as in Figure S6). As these cell lines were generally non-haploid, the multiple alleles were often present at unequal proportions.

We note that the tables list variants detected by the calling procedure in the PCR amplified regions. However, the Sanger chromatograms reveal more structure. For example, the experiment with MDAMB468 in gene ASMTL revealed additional variants that were filtered out by the thesaurus annotation due to an insertion (thesaurushard fileter code). This suggests that the annotation can be improved further to revela even more variants. As another example, Sanger sequencing in MDAMB157 in gene RASA4B showed evidence of variants that were completely absent in the read data.

Cell Line	Site	Gene	Primer Fw	Primer Rev
CAL51	chrX:1522190<br>chrY:1472190	ASMTL<br>ASMTL	CAGTCACGACTACACGCTCC	CCGTTCTGAGCTCCTGTTGC
MDAMB468	chrX:1531648<br>(chrY:1481648)	ASMTL<br>ASMTL	GCCCGGCCTCCTTGTAAAAC	CGGGCGTGACATCACTAACC
	chrX:1531700<br>(chrY:1481700)	ASMTL<br>ASMTL		
HCC1395	chrX:1537881<br>(chrY:1487881)	ASMTL<br>ASMTL	CCCATGATCTGGGACTCAGC	ACAAGAGTAGCCCACACAGC
	chrX:1537919<br>chrY:1487919	ASMTL<br>ASMTL		
	~~chrX:1537989~~<br>~~chrY:1487989~~	~~ASMTL~~<br>~~ASMTL~~		
MDAMB453	chrX:1553973<br>chrY:1503973	ASMTL<br>ASMTL	GCAGACTTCCAAATCGGCGG	TGCTGGTGGGATTTGTGACC
HCC1428	chrX:1557993<br>(chrY:1507993)	ASMTL<br>ASMTL	TTAGGCCCCTCATCTGCTGG	TGAGATGCTCCGTGAGTGCC
	chrX:1558072<br>(chrY:1508072)	intronic<br>intronic		
ZR751	chrX:1561089<br>(chrY:1511089)	ASMTL<br>ASMTL	GCTGTGCCACCAACTGCATC	AGGGAAAGCTTGGCGGATGG

Table S7: **Sanger validation in gene ASMTL in breast cancer cell lines.** Blocks of lines separated by lines indicate called variants within the PCR product amplified by the primers shown. Smaller blocks separated by white space group clusters of variants linked by thesaurus annotation. Sites in parentheses correspond to positions in dbSNP. Crossed out entries represent variants that were not visible in Sanger chromatograms.

Cell Line	Site	Gene	Primer Fw	Primer Rev
MDAMB468	chr7:102143669	RASA4B	ATGTCCCCTCAGAGAGGTCG	GAGATGGCTATGGGAGCAGG
	chr7:102242830	RASA4		
	chr7:102143808	RASA4B		
	chr7:102242969	RASA4		
	chr7:102143809	RASA4B		
	chr7:102242970	RASA4		
HCC1954	chr7:102128952	RASA4B	GCATTTCCCAAACCACCTGG	ATGTTCCACTGGAGGAAGGG
	chr7:102228124	RASA4B		
	chr7:102327203	intergenic		
MDAMB157	chr7:102141519	intronic	GTCCCTCTTTGGGGAAGCCG	ACCTCTGCTGTGGCAGAACC
	chr7:44005839	intronic		
	chr7:102240680	intronic		
	chr7:102141562	RASA4B		
	chr7:44005882	POLR2J4		
	chr7:102240723	RASA4		
	~~chr7:102141570~~	~~RASA4B~~		
	~~chr7:44005890~~	~~POLR2J4~~		
	~~chr7:102240731~~	~~RASA4~~		
HCC202	chr7:102141517	intronic	GTCCCTCTTTGGGGAAGCCG	ACCTCTGCTGTGGCAGAACC
	chr7:44005837	intronic		
	chr7:102240678	intronic		
	chr7:102141570	RASA4B		
	chr7:44005890	POLR2J4		
	chr7:102240731	RASA4		
	~~chr7:102141617~~	~~RASA4B~~		
	~~chr7:44005937~~	~~POLR2J4~~		
	~~chr7:102240778~~	~~RASA4~~		
MDAMB157	~~chr7:102135744~~	~~RASA4B~~	GGAACAGTGGCTCTCACAGC	TGCCCTGCATGTGTACCTGG
	~~chr7:102234913~~	~~RASA~~		
	~~chr7:102330823~~	~~intergenic~~		
	(chr7:102135806)	RASA4B		
	(chr7:102234975)	RASA4		
	(chr7:102330885)	intergenic		

Table S8: **Sanger validation of variants in gene RASA4B breast cancer cell lines.** Analogous to table S7 but showing variants in gene RASA4B.

References

[1] B. Langmead and S. L. Salzberg, "Fast gapped-read alignment with Bowtie 2," *Nature Methods*, vol. 9, no. 4, pp. 357–359, 2012.

[2] T. D. Wu and S. Nacu, "Fast and SNP-tolerant detection of complex variants and splicing in short reads," *Bioinformatics*, vol. 26, no. 7, pp. 873–881, 2010.

[3] A. McKenna, M. Hanna, E. Banks, A. Sivachenko, K. Cibulskis, A. Kernytsky, K. Garimella, D. Altshuler, S. Gabriel, M. Daly, *et al.*, "The genome analysis toolkit: a MapReduce framework for analyzing next-generation DNA sequencing data," *Genome Research*, vol. 20, no. 9, pp. 1297–1303, 2010.

[4] D. C. Koboldt, Q. Zhang, D. E. Larson, D. Shen, M. D. McLellan, L. Lin, C. A. Miller, E. R. Mardis, L. Ding, and R. K. Wilson, "VarScan 2: somatic mutation and copy number alteration discovery in cancer by exome sequencing," *Genome Research*, vol. 22, no. 3, pp. 568–576, 2012.

[5] ENCODE Project Consortium and others, "An integrated encyclopedia of dna elements in the human genome," *Nature*, vol. 489, no. 7414, pp. 57–74, 2012.

[6] A. Daemen, O. L. Griffith, L. M. Heiser, N. J. Wang, O. M. Enache, Z. Sanborn, F. Pepin, S. Durinck, J. E. Korkola, M. Griffith, *et al.*, "Modeling precision treatment of breast cancer," *Genome Biol*, vol. 14, no. 10, p. R110, 2013.

[7] 1000 Genomes Project Consortium and others, "A map of human genome variation from population-scale sequencing," *Nature*, vol. 467, no. 7319, pp. 1061–1073, 2010.

[8] D. Fimereli, V. Detours, and T. Konopka, "Triagetools: tools for partitioning and prioritizing analysis of high-throughput sequencing data," *Nucleic Acids Research*, p. gkt094, 2013.
