# Supplementary material for: A thesaurus of genetic variation for interrogation of repetitive genomic regions
Source: Nucleic Acids Res. 2015 Mar 27;43(10):e68. doi: 10.1093/nar/gkv178 (PMC4446415; doi:10.1093/nar/gkv178)
Supplement: SUPPLEMENTARY DATA [file supp_gkv178_nar-03253-met-n-2014-File006.docx]

# ADDITIONAL FILES

Additional file 1 – Supplementary Text

The Supplementary text contains details on calculations beyond the descriptions in the main text.
